# Knowledge of the risks associated with being underweight and body shape differences among young Japanese women: a cross-sectional study

**DOI:** 10.1186/s13030-025-00338-8

**Published:** 2025-10-02

**Authors:** Mariko Ogawa, Michiko Nakazato, Jinko Yokota, Kaori Koga

**Affiliations:** 1 Fukushima Medical Center for Children and Women, Fukushima Medical University, 1, Hikarigaoka, Fukushima City, 960-1295 Fukushima Japan; 2 Department of Psychiatry, School of Medicine, International University of Health and Welfare, 4-3, Kozunomori, Narita City, Chiba Japan; 3 Health Care Center, Tokyo Women’s Medical University, 8-1, Kawada-cho, Shinjuku-ku, Tokyo, Japan; 4 Department of Obstetrics and Gynecology, Reproductive Medicine, Graduate School of Medicine, Chiba University, 1−8−1Inohana Chuo-ku, Chiba City, Chiba Japan

**Keywords:** Female, Body weight, Body image, Eating disorders, Knowledge, Risk, Women’s health

## Abstract

**Background:**

In Japan, approximately 20% of young women are underweight, a rate higher than that of other developed countries. For women, being underweight at a young age has been associated with amenorrhea, eating disorders, osteoporosis, and adverse pregnancy outcomes. We investigated young women’s knowledge of these risks and associated factors.

**Methods:**

A web-based survey was conducted among 984 Japanese women aged 18–29 years. The survey included questions about actual body weight, perceived healthy weight, body image, eating disorder tendency, knowledge of various risks and factors associated with being underweight, and sources of this knowledge. Participants were divided into underweight (< 18.5 kg/m^2^), normal weight (18.5–25 kg/m^2^), and obese (≥ 25 kg/m^2^) groups based on their body mass index (BMI). The body image and knowledge of health risks associated with being underweight were compared across the three groups and with women with and without an eating disorder tendency.

**Results:**

Among the participants, 31.5% were underweight (BMI < 18.5 kg/m^2^). Of these, 87.4% considered their subjective ideal weight to be underweight, and 66.1% viewed their subjective healthy weight similarly. Underweight women reported greater body satisfaction than did those in other body shape groups. While 73.2% recognized amenorrhea as a risk of being underweight, only approximately half identified infertility, eating disorders, and osteoporosis as risks and associated factors, and few were aware of adverse pregnancy outcomes. Knowledge levels did not differ between underweight and normal-weight women. Conversely, women with a tendency toward an eating disorder were more aware of the risks of osteoporosis (58.6% vs. 49.0%) and eating disorders (66.8% vs. 55.2%) than were women without a tendency toward an eating disorder.

**Conclusions:**

Young Japanese women had insufficient knowledge about the adverse pregnancy outcomes associated with being underweight. Underweight women are not less aware of the health risks and associated factors associated with underweight than normal-weight women. The body shape of young women may not be influenced by their knowledge of health issues associated with being underweight.

## Background

Maintaining a healthy BMI has been associated with better overall health in adults. Studies have shown a J-shaped relation between BMI and all-cause mortality, with the lowest risk observed at a BMI of 20.0–24.9 [1]. However, a recent survey of 200 countries reported that, in 81% of them, the combined prevalence of obesity and underweight among women has increased [2]. In many developed countries, rising obesity rates are particularly concerning [2]. In Japan, approximately 30% of men are obese, raising concerns about cardiovascular disease [3]. Conversely, the high prevalence of underweight young women is a major public health concern: the 2022 National Health and Nutrition Survey reported that 19.1% of women in their 20s are underweight, a rate that has hovered around 20% for the past decade [4]. Japan is the only high-income country with such a high prevalence of underweight women [2].

Being underweight at a young age increases the risk of several health problems, including amenorrhea [5], infertility [6], and complications during pregnancy and childbirth [7, 8]. Recent research has also shown that poor maternal nutrition during pregnancy is linked to the development of lifestyle-related diseases in the child [9]. Additionally, inadequate weight during youth reduces bone mass, which increases the risk of osteoporosis and fractures later in life [10]. Eating disorders result from a complex interplay of factors [11] and occur more frequently in young women. The desire to be thin and the persistence of weight-loss behaviors are associated with the development and maintenance of eating disorders [12].

Efforts to prevent underweight and eating disorders, particularly in schools, have shown beneficial outcomes [13–15]. However, most of these reports have focused on short-term effects, with limited research into whether knowledge about the risks and factors associated with underweight impacts body weight and shape in real-world settings.

We hypothesized that knowledge of the risks and factors associated with underweight could affect the perception of current and ideal body shape. To explore this, we conducted an online survey to investigate young Japanese women’s perception of healthy body weight, their knowledge of the risks and factors associated with underweight, and their sources of this information. We also analyzed the differences in knowledge across body types.

## Methods

### Participants and study design

A web-based, self-administered questionnaire survey was conducted from September 26–27, 2024. Japanese women aged 18–29 years were randomly selected from a web-based panel at Cross Marketing, Inc., Japan, with an even distribution of participants across age groups (83–84 participants per group). Inclusion criteria were Japanese women who had lived in and been educated in Japan. Exclusion criteria were women with a history of childbirth, because childbirth may alter body image [16], and those who provided implausible height or weight responses.

The details of the questionnaire are provided later. The participants were divided into three groups based on their body mass index (BMI) in accordance with the Japanese Society for the Study of Obesity guidelines [17]: underweight group (< 18.5 kg/m^2^), normal-weight group (18.5–25 kg/m^2^), and obese group (≥ 25 kg/m^2^). The responses were compared among the three groups.

### Questionnaire

The web survey items were determined after discussions with the research team and included questions about demographic characteristics, current height and weight, subjective ideal body weight, perceived healthy body weight, body image, body satisfaction, knowledge of the risk and factors associated with underweight, and the sources of this knowledge. The survey also included the Sick, Control, One Stone, Fat, and Food (SCOFF) questionnaire to assess the possibility of an eating disorder. The SCOFF questionnaire is a brief, five-item screening tool for eating disorders specifically developed for use by non-specialists in primary care settings [18]. The Japanese version of the SCOFF questionnaire has been validated [19] and is widely used. Two or more positive responses on the SCOFF questionnaire suggest the possibility of an eating disorder and the need for further evaluation. In addition to comparing the three body shape groups, we divided all subjects into SCOFF-positive (two or more positive items) and SCOFF-negative groups.

### Statistical analysis

BMI was calculated as weight (kg) divided by height squared (m^2^). Participant characteristics were summarized using means for continuous variables and proportions for categorical variables. Data from the underweight, normal weight, and obese groups were compared. A comparison of knowledge regarding the risks and factors associated with underweight was also made of the SCOFF-positive and SCOFF-negative groups. Categorical variables were analyzed using the chi-squared test with Bonferroni-adjusted post hoc comparisons. Continuous variables that violated the assumption of homogeneity of variance were analyzed using Welch’s ANOVA, followed by Games-Howell post hoc tests. Statistical significance was set at *P* < 0.05. All analyses were performed using SPSS version 29.0 (IBM Corp., Armonk, NY, USA).

### Ethics

All participants were informed of the purpose of the study and provided consent before completing the questionnaire, which included questions on body shape and eating habits. This study was conducted in accordance with the principles of the Declaration of Helsinki. The Ethics Committee of Fukushima Medical University determined that an ethical review was not required for this study (REC2024-094).

## Results

**Fig. 1:**
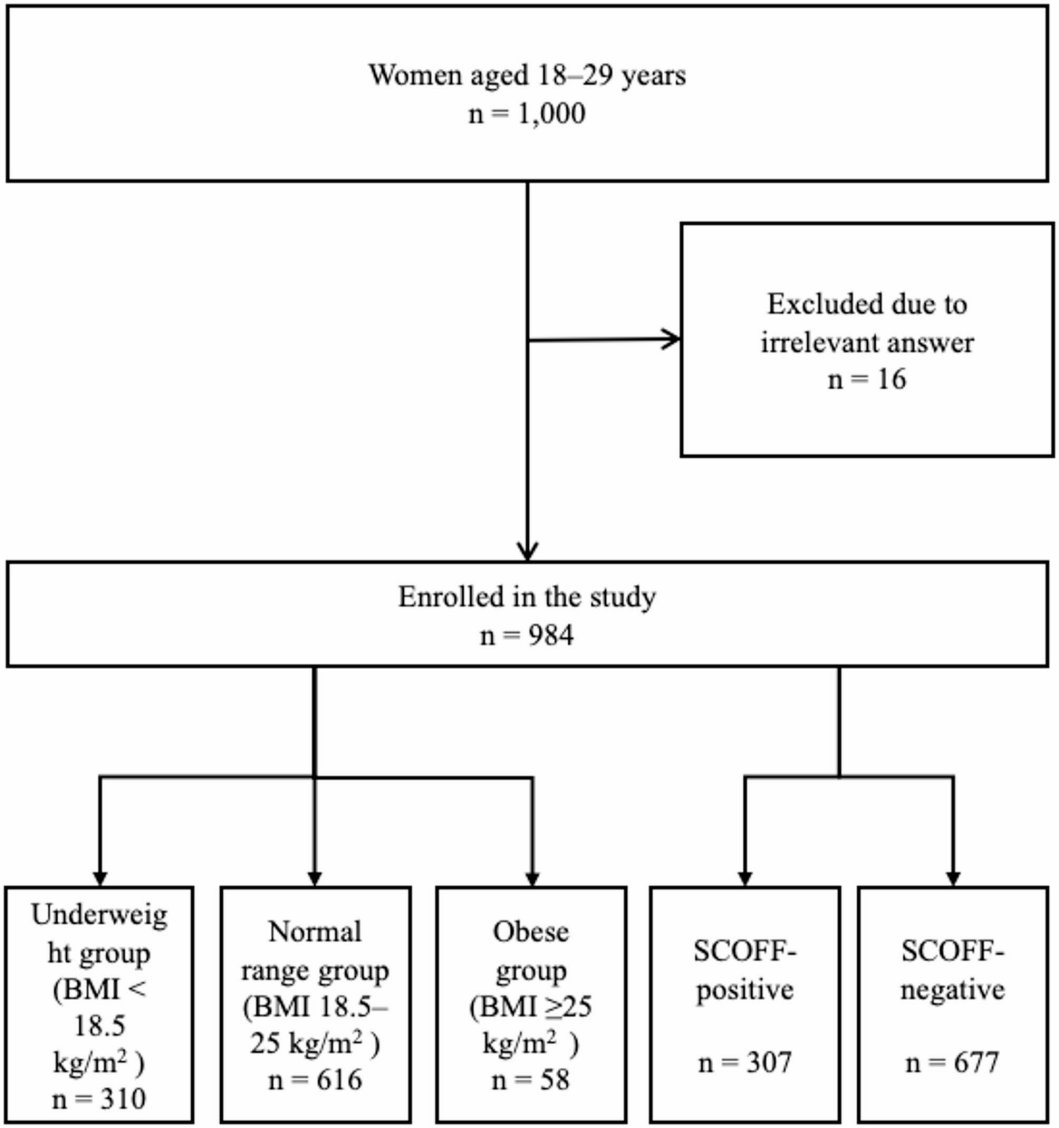
Flowchart of the dynamics of participant selection

A flow diagram of participant enrollment is shown in Fig. 1. Out of 1,000 participants who responded to the web survey, 16 were excluded because of inaccurate height and weight information, leaving the data of 984 available for analysis. The demographic characteristics of the study population in each group are presented in Table 1. Among the participants, 31.5% were underweight (BMI < 18.5), 62.5% were of normal weight (18.5 ≤ BMI < 25), and 5.9% were obese (25 ≤ BMI).A higher proportion of the obese group were single in comparison with the underweight group. Additionally, the obese group had a greater number of unemployed individuals, fewer were living with their parents, and they had a lower annual income than tthe normal-weight group. No significant differences were observed among the groups in terms of educational level or hormone therapy use.

**Table 1:** Demographic characteristics of the participants in each study group (*n* = 984)

	All (*n* = 984)		Underweight group (*n* = 310)		Normal weight group (*n* = 616)		Obese group (*n* = 58)		*P*-value
	n	%	n	%	n	%	n	%	
**Age**,** years**									
18–19	165	16.8	52	16.8	103	16.7	10	17.2	0.265
20–21	163	16.6	56	18.1	91	14.8	16	27.6	
22–23	165	16.8	53	17.1	108	17.5	4	6.9	
24–25	162	16.5	53	17.1	103	16.7	6	10.3	
26–27	163	16.6	45	14.5	107	17.4	11	19	
28–29	166	16.9	51	16.5	104	16.9	11	19	
**Marital status**									
Single	897	91.2	278_a_	89.7	561_a, b_	91.1	58_b_	100.0	0.039
Married	87	8.8	32_a_	10.3	55_a, b_	8.9	0_b_	0.0	
**Living situation**									
Alone	324	32.9	89_a_	28.7	223_a_	36.2	12_a_	20.7	0.007
With guardians	522	53.0	177_a, b_	57.1	305_b_	49.5	40_a_	69.0	
With a partner	117	11.9	36_a_	11.6	78_a_	12.7	3_a_	5.2	
Others	21	2.1	8_a_	2.6	10_a_	1.6	3_a_	5.2	
**Occupation**									
Student	290	29.5	97_a_	31.3	178_a_	28.9	15_a_	25.9	0.032
Worker	541	55.0	170_a_	54.8	344_a_	55.8	27_a_	46.6	
Housewife	14	1.4	4_a_	1.3	10_a_	1.6	0_a_	0.0	
Unemployed	104	10.6	35_a, b_	11.3	57_b_	9.3	12_a_	20.7	
Others	35	3.6	4_a_	1.3	27_b_	4.4	4_b_	6.9	
**Education level**									
< High school graduate	61	6.2	19	6.1	36	5.8	6	10.3	0.094
High school graduate	291	29.6	87	28.1	178	28.9	26	44.8	
>High school graduate	631	64.1	204	65.8	401	65.1	26	44.8	
Others	1	0.1	0	0.0	1	0.2	0	0.0	
**Income (1**,**000 yen/year)**									
< 2,000	586	59.6	190_a, b_	61.3	351_b_	57.0	45_a_	77.6	**0.042**
2000 ≤, < 6,000	368	37.4	111_a, b_	35.8	245_b_	39.8	12_a_	20.7	
6,000 ≤	30	3.0	9_a_	2.9	20_a_	3.2	1_a_	1.7	
**History of pregnancy**									
No	953	96.8	302	97.4	594	96.4	57	98.3	0.584
Yes	31	3.2	8	2.6	22	3.6	1	1.7	
**Hormone therapy**									
No	768	78.0	252	81.3	468	76.0	48	82.8	0.354
Oral contraceptives	155	15.8	44	14.2	105	17.0	6	10.3	
Other than oral contraceptives	11	1.1	4	1.3	6	1.0	1	1.7	
Don’t know	50	5.1	10	3.2	37	6.0	3	5.2	

BMI, body mass index

_a_, _b_, _c_ Values with different superscript letters are significantly different

Table 2 presents the actual, ideal, and perceived healthy BMIs for each group. The ideal BMI for the underweight group was 17.13 kg/m^2^ [95% confidence interval (CI): 16.93, 17.33], falling within the underweight range, and was reported by 87.4% of the participants. The perceived healthy BMI for the underweight group was 18.11 kg/m^2^ (95% CI: 17.93, 18.29), with 66.1% of the participants considering an underweight BMI to be healthy. Significant differences in the ideal and perceived healthy BMI were observed across the groups (*P* < 0.001).

**Table 2:** Actual,** ideal**,** and considered-to-be-healthy BMIs of each study group** n (%) or mean (SD), 95% CI

	All (*n* = 984)	Underweight group (*n* = 310)	Normal weight group (*n* = 616)	Obese group (*n* = 58)	*P*-value
**Actual body weight (BMI)**	19.97 (3.54), 19.75–20.19	16.92 (1.69), 16.74–17.11	20.64 (1.61), 20.51–20.77	29.14 (5.64), 27.66–30.63	
**Ideal body weight (BMI)**	18.58 (2.28), 18.43–18.72	17.13 (1.79) _a_, 16.93–17.33	18.94 (1.51) _b_, 18.83–19.07	22.41 (4.45) _c_, 21.24–23.58	< 0.001
Underweight (< 18.5)	511 (51.9%)	271_a_ (87.4%)	235_b_ (38.1%)	5_c_ (8.6%)	< 0.001
Normal range (18.5 ≤ and < 25)	468 (47.6%)	38_a_ (12.3%)	381_b_ (61.9%)	49_c_ (84.5%)	
Obese (25 < )	5 (0.5%)	1_a_ (0.3%)	0_a_ (0.0%)	4_b_ (6.9%)	
**Healthy body weight (BMI)**	19.31 (2.16), 19.18–19.45	18.11 (1.62) _a_, 17.93–18.29	19.61 (1.60) _b_, 19.48–19.73	22.72 _c_ (4.27), 21.59–23.84	< 0.001
Underweight (< 18.5)	322 (32.7%)	205_a_ (66.1)	115_b_ (18.7%)	2_c_ (3.4%)	< 0.001
Normal range (18.5 ≤ and < 25)	658 (66.9%)	105_a_ (33.9%)	501_b_ (81.3%)	52_b_ (89.7%)	
Obese (25 < )	4 (0.4%)	0_a_ (0.0%)	0_a_ (0.0%)	4_b_ (6.9%)	

SD, standard deviation; CI, confidence interval; BMI, body mass index

_a_, _b_, _c_ Values with different superscript letters are significantly different

The body image, body satisfaction, current dieting behavior, and eating disorder tendencies of each group are shown in Table 3. Among underweight women, 65.5% perceived themselves as ‘slightly underweight’ or ‘underweight,’ while 29% considered their weight “normal.” In the normal-weight group, 49.7% perceived themselves as normal weight, while 36.8% perceived themselves as ‘slightly overweight’ or ‘overweight.’

**Table 3:** Body image, body satisfaction, current dieting behavior, and eating disorder tendency in each study group

	All (*n* = 984)		Underweight group (*n* = 310)		Normal weight group (*n* = 616)		Obese group (*n* = 58)		*P*-value
	n	%	n	%	n	%	n	%	
**What do you think of your body shape?**									
Underweight	103	10.5	91_a_	29.4	12_b_	1.9	0_b_	0.0	< 0.001
Slightly underweight	183	18.6	112_a_	36.1	71_b_	11.5	0_c_	0.0	
Normal	398	40.4	90_a_	29.0	306_b_	49.7	2_c_	3.4	
Slightly overweight	219	22.3	13_a_	4.2	185_b_	30.0	21_b_	36.2	
Overweight	81	8.2	4_a_	1.3	42_b_	6.8	35_c_	60.3	
**Are you satisfied with your body shape?**									
Satisfied	144	14.6	66_a_	21.3	76_b_	12.3	2_b_	3.4	< 0.001
Somewhat dissatisfied	393	39.9	127_a_	41.0	252_a_	40.9	14_b_	24.1	
Dissatisfied	328	33.3	72_a_	23.2	218_b_	35.4	38_c_	65.5	
Can’t say either way	119	12.1	45_a_	14.5	70_a_	11.4	4_a_	6.9	
**Current dieting behavior**									
Yes	431	43.8	98_a_	31.6	305_b_	49.5	28_b_	48.3	< 0.001
No	553	56.2	212_a_	68.4	311_b_	50.5	30_b_	51.7	
**Eating disorder tendency (SCOFF score)**									
Yes ( 2 ≤ )	307	31.2	93_a_	30.0	187_a_	30.4	27_b_	46.6	0.034
No (< 2 )	677	68.8	217_a_	70.0	429_a_	69.6	31_b_	53.4	

SCOFF, the Sick, Control, One Stone, Fat, and Food questionnaire

_a_, _b_, _c_ Values with different superscript letters are significantly different

Regarding body satisfaction, 23.2% of the underweight group reported being dissatisfied, which is significantly lower than the 35.4% of the normal-weight group and 65.5% of the obese group.

There were fewer current dieting attempts in the underweight group than in the normal-weight and obese groups. Additionally, an eating disorder tendency (SCOFF-positive) was identified in 31.2% of the total sample, 30.0% of the underweight group, 30.4% of the normal-weight group, and 46.6% of the obese group. No significant differences were found between the underweight and normal-weight groups in terms of eating disorder tendency. A high tendency toward an eating disorder was observed in the obese group.

Table 4 compares awareness of underweight-associated risks and its associated factors across BMI categories. Overall, 73.2% recognized amenorrhea as a consequence of being underweight, but awareness of pregnancy complications and fetal health impacts was limited. Knowledge did not differ between the underweight and normal-weight groups. However, the obese group was less aware than the underweight group of the risk of infertility. Knowledge of the risk of preterm birth was lower in the obese group than in the normal-weight group.

**Table 4:** Comparison of knowledge about body shape and the risks associated with being underweight

	All (*n* = 984)		Underweight group (*n* = 310)		Normal weight group (*n* = 616)		Obese group (*n* = 58)		*P*-value
	n	%	n	%	n	%	n	%	
Method of calculating proper body weight	458	46.5	160_a_	51.6	277_a_	45.0	21_a_	36.2	0.043
If you are underweight, your period may stop.	720	73.2	230	74.2	450	73.1	40	69.0	0.707
Being underweight can cause infertility.	475	48.3	164_a_	52.9	292_a, b_	47.4	19_b_	32.8	0.015
Thin women are more likely to have premature births during pregnancy.	255	25.9	83_a, b_	26.8	165_b_	26.8	7_a_	12.1	0.046
Thin women are more likely to give birth to low birth weight babies.	314	31.9	102	32.9	198	32.1	14	24.1	0.413
Babies born to underweight mothers are more likely to develop lifestyle diseases such as diabetes in the future.	160	16.3	52	16.8	105	17.0	3	5.2	0.062
People who are underweight and restricting their diet may develop an eating disorder.	579	58.8	189	61.0	354	57.5	36	62.1	0.520
People who are thin are more likely to develop osteoporosis and suffer from bone fractures.	512	52.0	159	51.3	317	51.5	36	62.1	0.288

Number of participants responding “I know.”

_a_, _b_, _c_ Values with different superscript letters are significantly different

Table 5 compares the knowledge of risks and factors associated with underweight between the SCOFF-positive and SCOFF-negative groups. The SCOFF-positive group, with a tendency toward an eating disorder, was more aware of eating disorders and osteoporosis as factors associated with being underweight than was the SCOFF-negative group. 58.6% of the SCOFF-positive group was aware of the osteoporosis risk compared to 49.0% of the SCOFF-negative group.

**Table 5:** Comparison of knowledge about the risks and factors associated with underweight based on the SCOFF results

	SCOFF-positive (*n* = 307)		SCOFF-negative (*n* = 677)		*P*-value
	*n*	%	*n*	%	
Method of calculating proper body weight	149	48.5	309	45.6	0.409
If you are underweight, your period may stop.	227	73.9	493	72.8	0.756
Being underweight can cause infertility.	150	48.9	325	48.0	0.836
Thin women are more likely to have premature births during pregnancy.	79	25.7	176	26.0	1.000
Thin women are more likely to give birth to low-birth-weight babies.	93	30.3	221	32.6	0.507
Babies born to underweight mothers are more likely to develop lifestyle diseases such as diabetes in the future.	48	15.6	112	16.5	0.780
People who are underweight and restricting their diet may develop an eating disorder.	205	66.8	374	55.2	0.000
People who are thin are more likely to develop osteoporosis and suffer from bone fractures.	180	58.6	332	49.0	0.006

Number of participants responding “I know.”

SCOFF, the Sick, Control, One Stone, Fat, and Food questionnaire. SCOFF-positive: 2 ≤

Finally, we asked the participants where they learned about the risks and factors associated with underweight (Table 6, 7). School was the most frequently reported source of information on amenorrhea, infertility, and pregnancy complications. Although information on fetal health risks was primarily acquired in college, knowledge of other risks was most often gained during junior high school. Conversely, the Internet and social networking sites were the most common sources for learning how to calculate a proper body weight. Television and magazines were the most frequently cited sources of information on eating disorders as factors associated with underweight.

**Table 6:** Source of information on body shape and risks of being underweight (multiple answers possible)

		Education						Information from						
	n†	Up to junior high school		High school		College		Guardian		Friend		Specialist		
		n	%	n	%	n	%	n	%	n	%	n	%	
Method of calculating proper body weight	458	84	18.3	63	13.8	46	10.0	11	2.4	17	3.7	7	1.5	
If you are underweight, your period may stop.	720	**183**	**25.4**	115	16.0	41	5.7	64	8.9	23	3.2	18	2.5	
Being underweight can cause infertility.	475	**97**	**20.4**	62	13.1	42	8.8	34	7.2	9	1.9	18	3.8	
Thin women are more likely to have premature births during pregnancy.	255	**41**	**16.1**	32	12.5	30	11.8	11	4.3	7	2.7	7	2.7	
Thin women are more likely to give birth to low birth weight babies.	314	**53**	**16.9**	**53**	**16.9**	48	15.3	20	6.4	6	1.9	21	6.7	
Babies born to underweight mothers are more likely to develop lifestyle diseases such as diabetes in the future.	160	18	11.3	26	16.3	**34**	**21.3**	6	3.8	2	1.3	5	3.1	
People who are underweight and restricting their diet may develop an eating disorder.	579	72	12.4	64	11.1	49	8.5	43	7.4	24	4.1	15	2.6	
People who are thin are more likely to develop osteoporosis and suffer from bone fractures.	512	**118**	**23.0**	68	13.3	44	8.6	42	8.2	8	1.6	17	3.3	

†: Total number of “I know”

Bold numbers indicate the most frequent response, excluding “unclear”

**Table 7:** Source of information on body shape and risks of being underweight (multiple answers possible)—continued

	*n*†	Internet information				Television and magazines		Social media		Others		Unclear	
		Medical institution website		Other than medical institutions									
		*n*	%	*n*	%	*n*	%	*n*	%	*n*	%	*n*	%
Method of calculating proper body weight	458	27	5.9	**115**	**25.1**	27	5.9	**115**	**25.1**	1	0.2	88	19.2
If you are underweight, your period may stop.	720	56	7.8	130	18.1	82	11.4	145	20.1	14	1.9	153	21.3
Being underweight can cause infertility.	475	40	8.4	74	15.6	56	11.8	94	19.8	2	0.4	115	24.2
Thin women are more likely to have premature births during pregnancy.	255	21	8.2	34	13.3	26	10.2	40	15.7	1	0.4	71	27.8
Thin women are more likely to give birth to low birth weight babies.	314	48	15.3	42	13.4	42	13.4	48	15.3	11	3.5	3	1.0
Babies born to underweight mothers are more likely to develop lifestyle diseases such as diabetes in the future.	160	14	8.8	19	11.9	12	7.5	16	10.0	0	0.0	54	33.8
People who are underweight and restricting their diet may develop an eating disorder.	579	37	6.4	109	18.8	**162**	**28.0**	130	22.5	7	1.2	107	18.5
People who are thin are more likely to develop osteoporosis and suffer from bone fractures.	512	34	6.6	81	15.8	111	21.7	80	15.6	1	0.2	110	21.5

†: Total number of “I know”

Bold type indicates the most frequent response, excluding “unclear”

## Discussion

The prevalence of underweight in this study was 31.5%, exceeding the 20.2% reported for women in their 20–30 s in national surveys [4]. The obese group was primarily composed of single women with lower annual incomes and a higher proportion of unemployment in comparison with the normal-weight group. Associations between low annual income and unemployment and obesity have been reported previously [20, 21], and our results support these findings. Conversely, reports on the socioeconomic factors of underweight women are scarce. One report suggested that underweight women tend to have high parental education levels [22], but no socioeconomic differences were observed between the underweight and normal-weight women of this study.

Underweight women in our study regarded an underweight ideal body weight as healthy. A survey of Japanese university students reported that underweight women preferred maintaining their current weight [23], and another found that they had lower ideal body weights than normal-weight women [24], which is in alignment with our findings. Our results also suggest that underweight women view their current weight as healthy, which indicates that they think they do not need to increase their weight, either for an ideal or a healthy weight.

Although many underweight women in the present study recognized their status, they were more likely to express satisfaction with their body shape compared to women in the normal-weight and obese groups. Previous research on underweight women has yielded mixed findings regarding body satisfaction, with some studies reporting high satisfaction and others reporting dissatisfaction [25, 26]. Conversely, higher BMI has been correlated with lower body satisfaction among young Japanese women [27, 28], , and our results reflect this finding.

Approximately 30% of the participants screened were positive for an eating disorder, with a notably high prevalence in the obese group. A study of Mexican college students reported a 24.2% positive SCOFF rate among young women [29], while a Japanese university survey (including both men and women) found a 15.4% positive rate [19]. Our observed rate is high relative to these findings. A recent systematic review concluded that the SCOFF questionnaire is a useful screening tool for anorexia nervosa and bulimia nervosa in young women [30]. The exact prevalence of eating disorders among young Japanese women is unknown, but our results suggest that it may not be low.

Many participants recognized that being underweight can cause amenorrhea, and about half identified infertility, eating disorders, and osteoporosis as factors associated with being underweight. However, awareness of adverse pregnancy outcomes was generally low. Notably, underweight women were not less aware of underweight-associated factors than women in other BMI categories. Few studies have examined the relation between underweight status and awareness of associated factors. This study revealed that underweight women had similar knowledge to normal-weight women about factors associated with being underweight, although knowledge regarding pregnancy complications was insufficient across all body shape groups. We further compared knowledge levels based on the presence or absence of an eating disorder tendency and found that participants with an eating disorder tendency had more knowledge than other participants about eating disorders and osteoporosis: women with an eating disorder tendency may be more likely to be informed by others about the possibility of developing an eating disorder and osteoporosis. However, they may not perceive these as problems for themselves. A study of former and current patients with eating disorders showed that many of them were familiar with the concept of eating disorders, but did not think it applied to themselves [31], and our findings support to this. Longitudinal studies are needed to determine if knowledge of the factors associated with being underweight can help young women to prevent eating disorders.

We also investigated where participants learned about the risks and factors associated with being underweight. Many reported acquiring information on amenorrhea, infertility, pregnancy complications, and osteoporosis in school, with most knowledge gained by junior high. This indicates that formal education is a primary source. Conversely, information on ideal body weight was mainly obtained from the Internet and social media, whereas television and magazines were the leading sources for information on eating disorders. Japanese curriculum guidelines have recently incorporated eating disorders into health and physical education classes, so future responses may change. Appropriate education is known to play a crucial role in healthy weight control, particularly among overweight individuals [32]. However, it remains unclear whether risk education will prompt young underweight women to pursue healthy weight gain.

This study has some limitations. First, it relied on a web-based sample, which may limit generalizability. The high proportion of underweight respondents suggests potential sampling bias. Second, height and weight were self-reported, meaning the calculated BMI may not reflect actual body composition: weight tends to be underestimated, and height overestimated [33]. Third, the questionnaire on underweight risks and associated factors lacked validation, creating uncertainty about its adequacy for assessing knowledge. Finally, the cross-sectional design limits our ability to assess causality between knowledge and weight-related perceptions or behaviors. Future studies should use longitudinal designs to examine how knowledge and body image evolve over time. These studies should measure body weight and height and use validated risk-assessment questionnaires. Despite these limitations, this is the first study to assess knowledge of underweight risks and its associated factors among young Japanese women. We believe these findings will be a valuable resource for efforts to address young women’s desire for thinness.

## Conclusions

There was no evidence suggesting that underweight women were less aware than others of health problems associated with being underweight. Additionally, many women with an eating disorder tendency recognized the risk of osteoporosis associated with being underweight. Knowledge of health problems associated with being underweight does not appear to deter young Japanese women from maintaining an underweight status. Further research is needed to identify factors that promote behaviors aimed at preventing underweight.

## Data Availability

The datasets used and/or analyzed in the current study are available from the corresponding author upon reasonable request.

## Abbreviations

BMI: Body mass index
SCOFF: The Sick, Control, One Stone, Fat, and Food questionnaire
